# Limited Role of Rhamnolipids on Cadmium Resistance for an Endogenous-Secretion Bacterium

**DOI:** 10.3390/ijerph191912555

**Published:** 2022-10-01

**Authors:** Sufang Xing, Zhen Yan, Chao Song, Huifang Tian, Shuguang Wang

**Affiliations:** 1 Shandong Key Laboratory of Water Pollution Control and Resource Reuse, School of Environmental Science and Engineering, Shandong University, Qingdao 266237, China; 2 Sino-French Research Institute for Ecology and Environment (ISFREE), School of Environmental Science and Engineering, Shandong University, Qingdao 266237, China

**Keywords:** cadmium stress, endogenous rhamnolipids, biosurfactants characteristics, cytotoxicity reduction

## Abstract

Rhamnolipids, a type of biosurfactant, represent a potential strategy for both enhancing organismic resistance and in situ remediation of heavy metals contaminations. In-depth study of the mechanism of rhamnolipids synthesis in response to heavy metals stress, is indispensable for a wide use of biosurfactant-secreting microbes in bioremediation. In this study, we employed the wild-type and the *rhlAB* deficient strain (Δ*rhlAB*) of *Pseudomonas aeruginosa*, a prototypal rhamnolipids-producing soil microorganism, to investigate its responses to cadmium resistance based on its physicochemical, and physiological properties. Compared with the wild-type strain, the Δ*rhlAB* were more sensitive to Cd-stress at low Cd concentration (<50 mg/L), whereas there was little difference in sensitivity at higher Cd concentrations, as shown by spot titers and cell viability assays. Secreted rhamnolipids reduced intracellular Cd^2+^ accumulation to alleviate Cd^2+^ stress, whereas endogenous rhamnolipids played a limited role in alleviating Cd^2+^ stress. Synthesized rhamnolipids exhibited a higher critical micelle concentration (CMC) (674.1 mg/L) and lower emulsification index (4.7%) under high Cd-stress, while these parameters showed no obvious changes. High Cd-stress resulted in high hydrophilic wild-type bacterial surface and lower bioremediation ability. This study could advance a deeper understanding of the mechanism of cadmium resistance and provide a theoretical foundation for the application of biosurfactant and biosurfactant-secreted bacterium in contaminant bioremediation.

## 1. Introduction

Increasingly serious cadmium (Cd) pollution has been caused by industrialization and technological development with large amounts of waste released [1,2,3]. Heavy metals can cause non-biodegradable bioaccumulation and have remarkable toxicity to organisms, leading to severe damage to biology and ecosystems in low concentrations [4,5,6]. For example, cadmium (Cd) can accumulate in the food chain and be a cause of great public concern, such as the Cd-related disease (Itai-Itai disease) in Japan [7,8].

Several remediation technologies have been developed for heavy metal contaminants, such as precipitation, membrane filtration, electrochemistry, adsorption, and phytoremediation [9,10,11,12]. However, most of these have limited practical applications due to high costs, energy requirements, and toxic secondary pollutions [13,14]. As a cost-effective and green strategy, bioremediation through biological scavenging of heavy metals by microorganisms, can be considered as an attractive alternative to those physiochemical processes [15,16,17]. In general, bioremediation can be performed both in situ and ex situ strategies. Compared to ex situ strategies, in situ bioremediation is more economical and effective but seriously limited by the microbial inhibition of heavy metals. Therefore, it is very meaningful to enhance the microbial resistance to heavy metals during in situ bioremediation.

Biosurfactants can combine with metal ions and have been successfully utilized in the elimination of heavy metals in the environment [9,18]. As a kind of anionic glycolipid biosurfactant, rhamnolipids provide various coordination sites with a strong affinity for heavy metal-rhamnolipid vesicles, and thus modify the bioavailability and mobilization of heavy metals, leading to high removal efficiency [19,20,21]. It is worth noting that rhamnolipids could enhance microbial resistance to Cd^2+^. For example, minimal inhibition concentration of Cd^2+^ from 92 μg/L to 246 μg/L for *Escherichia coli* [22]. Moreover, rhamnolipids could preferentially bond with Cd^2+^ compared with other heavy metals (Cd = Cr > Pb = Cu > Ni) [23]. Rhamnolipids are generally performed in situ (secreted by microbe in contaminated sites) and ex situ (industrial production) strategies [10,18]. Compared to in situ strategies, ex situ rhamnolipids seem to have a higher economic cost due to their higher natural biodegradability. It is worth noting that in situ rhamnolipids have been treated as potential applications for amplifying microbial pollutants resistance.

As a ubiquitous microbe that secretes rhamnolipids, *Pseudomonas aeruginosa* is commonly used for in situ bioremediation, and it has also been reported to secrete rhamnolipids as secondary metabolites [24,25,26]. Moreover, *P. aeruginosa* presents a high resistance to many heavy metals, especially to Cd [27,28,29,30]. Therefore, *P. aeruginosa* would be an optimal microorganism for rhamnolipids synthesis and the in situ remediation of Cd pollution. However, both the physicochemical properties and the structures of rhamnolipids are closely related to the cultivation environment of *P. aeruginosa*, which directly affect its remediation capacity [23,31]. Previous studies has focused on rhamnolipid secretion and associated gene expression at different growth stages under heavy metal stress and the effect of exogenous rhamnolipid administration to alleviate heavy metal toxicity in plants and microorganisms [10,22,32,33]. However, the endogenous rhamnolipids’ response to Cd^2+^ stress has not been fully explored, especially in regard to bacterial physicochemical and biochemical properties.

This study aimed to elucidate the interactions among microbe-endogenous rhamnolipids-Cd, including the role of endogenous rhamnolipids on bacterial resistance and the impact of cadmium on rhamnolipids’ properties. To further investigate the response of rhamnolipids synthesis on Cd^2+^ stress, we constructed the Δ*rhlAB* (*rhlAB* deficiency) strain (no rhamnolipids secretion) via the single-step double-recombination approach [34]. We then examined the Cd^2+^ distribution, cell metabolism, and biological activity of the wild-type and the Δ*rhlAB* strains and the physicochemical properties of secreted rhamnolipids response to Cd^2+^ stress. This research provides important theoretical support for the wide applications of biosurfactant-secreted microbes in bioremediation.

## 2. Materials and Methods

### 2.1. Bacterial Strains and Growth Conditions

The strains and plasmids used in this study are listed in Supplementary Information Table S1. All bacteria were grown from single-colony isolates in the Luria–Bertani (LB) broth at 37 °C and 180 rpm. When required, antibiotics were added to the media at the following final concentrations: 60 μg/mL gentamicin (Gm) for *P. aeruginosa* and 20 μg/mL Gm for *E. coli* WM 3064. Then, 0.3 mM 2,6-diaminopimelic acid (DAP) was obtained for the growth of *E. coli* WM 3064.

### 2.2. Plasmid Construction and Gene Deletion Mutant

The biosynthesis of rhamnolipids mainly relates to rhamnosyltransferase-1 (RhlAB) encoded by *rhlAB* operon. RhlA is involved in the synthesis of fatty acids, and *rhlB* catalyzed the synthesis of mono-rhamnolipids [35,36]. In this study, we deleted *rhlAB* genes to cut off the synthetic pathways of rhamnolipids and obtained the Δ*rhlAB* strain that could not secrete rhamnolipids. Fragments of 400~500 bp gene for the upstream or downstream of the *rhlAB* were amplified by gene splicing overlap extension (SOE-PCR) (primers listed in Table S2) based on *P. aeruginosa* genomic as template DNA. The fragments of the desired mutant allele were inserted into the suicide plasmid pEX18GM (Figure S1). This resulting deletion vector was manipulated with *E. coli* DH5α, then transformed into the donor strain *E. coli* WM3064 (auxotroph) and mobilized into *P. aeruginosa* by conjugation [34]. The Δ*rhlAB* cells were selected through two selections (Gm resistance selection and counter-selected by 15% sucrose). The Δ*rhlAB* strain was confirmed by PCR, sequencing, and blue agar tests with no rhamnolipids secretion (details are described in Supplementary Information).

### 2.3. Detection Analysis of Rhamnolipids

In order to analyze rhamnolipids productions, the wild-type bacteria grown with different Cd^2+^ concentrations were collected in the stationary phase and centrifuged for 20 min at 10,000× *g* to remove bacterial cells. The supernatant liquids were filtered through 0.22 μm filter membranes twice to wipe out residual cells. The cell-free supernatant was acidified to pH ≈ 2 and incubated at 4 °C overnight to precipitate rhamnolipids. The precipitated rhamnolipids were removed via centrifugation (10,000× *g*, 20 min, 4 °C) and re-dissolved in sterilized deionized water and then extracted with chloroform–methanol (2:1, *v*/*v*). Semi-purified rhamnolipids were obtained via evaporating the organic phases in a rotary evaporator (RE-52AA, Shanghai Yarong Biochemistry Instrument Factory) at 50 °C. The harvested rhamnolipids were lyophilized into powder and stored at desiccator for the following experiments.

To quantifying rhamnolipids, the amounts of rhamnose moiety were first determined using a anthrone-sulfuric acid colorimetric assay with L-rhamnose as the standard substance [37]. The rhamnolipids concentrations were obtained via the multiplication of the values of rhamnose moiety amounts by 3.4, which was the coefficient of the relation between purified rhamnolipids and rhamnose [22,38].

### 2.4. Characteristics Analysis of Rhamnolipids

The obtained rhamnolipids were analyzed with surface tension measurement, emulsifying activity, critical micelle concentration (CMC), and FTIR-ATR [18]. Surface tension assays were performed at room temperature using a tension meter (JK99C, Shanghai, China). Emulsification activities were measured by emulsification index for five days. CMCs were determined with a breaking point of the surface tension versus a series of rhamnolipids concentrations. The details were described in the Supplementary Information.

### 2.5. Distribution of Cd on Bacterial Media

To ascertain Cd^2+^ processing ability, wild-type and Δ*rhlAB* of *P. aeruginosa* were grown in the LB media with a series of Cd^2+^ concentrations (5 and 200 mg/L). For each group, a supernatant was taken in the stationary phase and centrifuged at 5000 rpm for 10 min. The supernatant was collected to measure the amounts of supernatant Cd^2+^ ions (C_Sup_). The precipitate was washed three times with EDTA to remove the Cd^2+^ bonded to the bacterial surface, and the washed cells were collected by centrifugation to determine intracellular Cd^2+^ (C_B_). Meanwhile, the control group was also prepared without bacteria cells to determine the total Cd^2+^ (C_Total_). All samples were acids digested with HClO_4_:HNO_3_ (1:9), and then the amounts of Cd^2+^ were analyzed with an atomic fluorescence spectrophotometer (AFS-933, Beijing Jitian Instrument Co., Ltd., Beijing, China). The amounts of Cd^2+^ absorbed on the bacterial surfaces (C_Sur_) were calculated as follows:

C_Sur_ = C_Total_ − C_Sup_ − C_B_ (1)

The percentage of each part (C_Sup_, C_B_, and C_Sur_) was calculated by dividing the Cd^2+^ amount of each part by the C_Total_ and then multiplied by 100.

### 2.6. Bacterial Surface Properties Analysis

Bacterial hydrophobicity was determined with bacterial adherence to hydrocarbon tests (BATHs) [39]. Briefly, *P. aeruginosa* were cultured in the LB medium with different Cd^2+^ concentrations, and cells were collected in the stationary phase via centrifugation at 5000 rpm for 15 min and washed with NaCl solution (0.85%, pH = 7.2~7.4) three times. Then, bacterial pellets were resuspended with 0.85% NaCl solution and adjusted OD_600_ ≈ 0.5 as the OD_initial_. N-dodecane at equal volume was mixed with the suspension and vortexed for 90 s. The mixture was then placed at room temperature for 30 min, and the absorbance of the inorganic phase was measured at 600 nm as the OD_final_. The bacterial hydrophobicity was calculated with the following equation:

Hydrophobicity (%) = 100% × (1 − OD_final_/OD_initial_) (2)

Cellular morphology. Wild -type and Δ*rhlAB* of *P. aeruginosa* were cultured in the LB medium with different Cd^2+^ concentrations (0, 5, 200 mg/L Cd). The cells were harvested in the stationary phase and then fixed, dehydrated, and coated with gold film, followed by observation with a field emission scanning electron microscope (Quanta 250 FEG, FEI, Hillsboro, OR, USA).

### 2.7. Bacterial Viability Assays

Spot Titer Assays. Wild-type and Δ*rhlAB* strains of *P. aeruginosa* were harvested from LB media without Cd^2+^ and then resuspended with 0.85% NaCl solution to adjust OD_600_ to 1.0. Serial 5-fold dilutions (10^−2^ to 10^−7^ for 0 to 50 mg/L Cd, and 10^0^ to 10^−5^ for 100 and 200 mg/L Cd, respectively) of 2 μL bacterial suspensions were used on the LB-agar plates with a series of Cd^2+^ concentrations and then the plates were incubated at 37 °C overnight [40].

Colony-Forming Unit (CFU) assays. Bacterial cells were harvested in the stationary phase and resuspended with 0.85% NaCl solution contained different Cd^2+^ concentrations (5, 20, and 200 mg/L). Samples were taken at 0 h and 18 h for CFU assays, named CFU_0_ and CFU_18_, respectively. Bacterial viability was calculated as the percentage of living cells using Equation (3) as follows:

Bacterial viability = CFU_18_/CFU_0_ × 100% (3)

### 2.8. Bacterial Biochemical Characteristic

Oxidative stress assays. Two type strains of *P. aeruginosa* were harvested in the stationary phase, which were exposed to Cd^2+^ at different dosages (0, 5, and 200 mg/L). The cells were collected and resuspended with 0.85% NaCl solution to similar OD_600_. Reactive oxygen species (ROS), MDA, GSH, and ATPase were determined in order to explore different responses of cells to Cd stress. In brief, ROS were tested with ROS Assay Kits (Beyotime Institute of Biotechnology, Shanghai, China) using a microplate spectrophotometer (Spark, Tecan Austria GmbH, Salzburg, Austria) with an excitation wavelength of 488 nm and emission wavelength of 525 nm. The oxidative damage to cells (MDA) was estimated using the thiobarbituric acid reactive substances (TBARS) method [41]. ATPase activity, GSH, and total sulfhydryl (total-SH) assays were measured with ATPase activity test kits, reduced glutathione (GSH) assay kits, total sulfhydryl (-SH) measurement kits (Nanjing Jiancheng Bioengineering Institute, Nanjing, China), respectively. Bacterial total proteins were measured with the BCA measurement kits (Nanjing Jiancheng Bioengineering Institute, Nanjing, China).

### 2.9. Statistical Analysis

The data of Cd distribution, cells viability, bacterial surface hydrophobicity, and bacterial biochemical properties among different treatment were assessed by two-way AVOVA analysis (factor 1 = Cd concentration, factor 2 = bacterial type). The data concerning rhamnolipids yields and surface tension assays were evaluated by one-way ANOVA. The statistically significant differences of the data described as follows: (none) indicated no difference (*p* > 0.05), (*) denoted *p* < 0.05; (**) denoted *p* < 0.01, (***) denoted *p* < 0.005.

## 3. Results and Discussion

### 3.1. Effect of Cd^2+^ on Cells Viability

**Figure 1:**
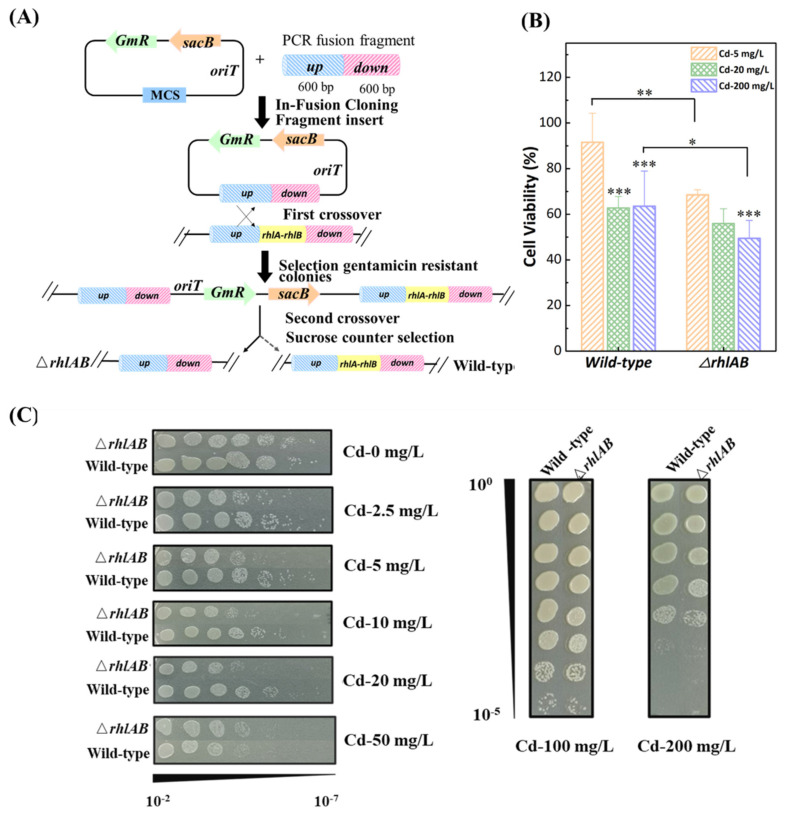
Construction of the Δ*rhlAB* strain using the two-step allelic exchange by pEX18GM (**A**), cell viability tests (**B**), and spot titer assays (**C**) of *P. aeruginosa* wild-type and Δ*rhlAB* strains under a series of Cd concentrations in the LB media. Differences were considered significant at *p* < 0.05, and * presented *p* < 0.05, ** presented *p* < 0.01, *** presented *p* < 0.005.

To explore the response of rhamnolipids to Cd^2+^ stress, the two-step allelic exchange approach was adopted to knockout the *rhlAB* gene of *P. aeruginosa,* and the Δ*rhlAB* strain was detected by PCR analysis, rhamnolipids secretion, and blue agar tests (Figure 1A and Figure S2).

Biological activities under Cd^2+^ stress were assessed by bacterial viability and spot titer to determine bacterial sensitivity to Cd^2+^. The Δ*rhlAB* strain showed lower bacterial viability (68.5%, 56.0%, and 49.4% for 5, 20, and 200 mg/L Cd, respectively) than wild-type (91.6%, 62.8%, and 63.6% for 5, 20, and 200 mg/L Cd, respectively) (Figure 1B). In addition, spot titer assays provided a visually observable indication of the sensitivity of cells to Cd^2+^ (Figure 1C). The Δ*rhlAB* strain presented as being more sensitive to low doses of Cd^2+^ (<50 mg/L) than the wild-type, which is consistent with bacterial viability tests. However, at high Cd^2+^ concentrations (>50 mg/L), slight differences in sensitivity were observed for two types of strains. Thus, rhamnolipids were able to enhance the bacterial resistance to Cd^2+^ at low doses, with limited increases at high Cd^2+^ levels.

### 3.2. Distribution of Cd^2+^ in Bacteria

**Figure 2:**
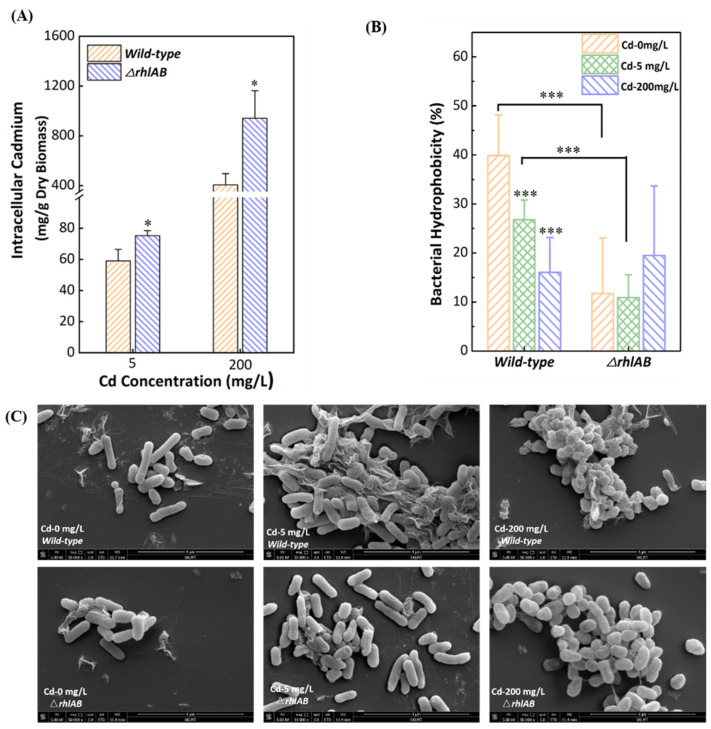
Intracellular cadmium amount per dry biomass (**A**), bacterial hydrophobicity (**B**), and SEM images (**C**) for wild-type and Δ*rhlAB* strains exposed to a series of Cd concentrations. Differences were considered significant at *p* < 0.05, and * presented *p* < 0.05, *** presented *p* < 0.005.

To investigate the effect of rhamnolipid synthesis on bacterial susceptibility to Cd^2+^, the distributions of Cd^2+^ on the surface, inside the cell, and in the supernatant of the two strains were determined (Figure 2A and Figure S3). Cadmium was mainly distributed on the bacterial surface of the two strains. The percentage of cadmium increased significantly from 48.9% to 62.1% (5 mg/L and 200 mg/L Cd^2+^, respectively) on the wild-type bacterial surfaces but remained stable (52.1% and 51.4%) in the Δ*rhlAB* cells. As a result, more intracellular Cd^2+^ was detected in Δ*rhlAB* cells than in wild-type strains. We evaluated intracellular Cd^2+^ per dry weight to eliminate the effect of bacterial biomass on Cd accumulation. As shown in Figure 2B, the amounts of Cd^2+^ were 59.1 mg/g and 406.1 mg/g in the wild-type strain while those were 75.3 mg/g and 1025.8 mg/g in the Δ*rhlAB* strain. These results suggested that mutant cells take up more Cd^2+^ into the cells, so rhamnolipids might play a significant role in blocking the entry of Cd^2+^ into the cells. This is mainly because rhamnolipids form a complex with Cd^2+^ rapidly within 15 min and remain stable for at least 27 h [42]. The morphologies of two strains with and without Cd^2+^ treatment were shown in SEM images (Figure 2C). They revealed that the Δ*rhlAB* strain looked fuller than wild-type cells at high Cd^2+^ concentration, which might be related to the large amount of Cd^2+^ entering the cells. Rhamnolipids combined with extracellular Cd^2+^ effectively reduced intracellular Cd^2+^ accumulation.

### 3.3. Effect of Cd^2+^ on Bacterial Characteristics

Cadmium exposure altered cell-surface properties. As shown in Figure 2B, the hydrophobicity of the wild-type was higher than that of the Δ*rhlAB* strain and decreased with increasing Cd^2+^ concentration, whereas that of the Δ*rhlAB* strain remained stable. This is mainly due to the role of rhamnolipids. Rhamnolipids are secreted around the cells and thus alter the hydrophobicity of the bacterial surface [43].

Since Cd^2+^ inhibited bacterial growth and rhamnolipids alleviated the negative effects of Cd^2+^ on cells, it seemed reasonable to assume that Cd^2+^ stress disrupted the metabolism in *P. aeruginosa*, including the biosynthesis and secretion strategy of endogenous rhamnolipids. To prove this, we examined the response of *P. aeruginosa* to Cd^2+^ stress.

### 3.4. Response of Antioxidant Systems to Cd^2+^ Stress

**Figure 3:**
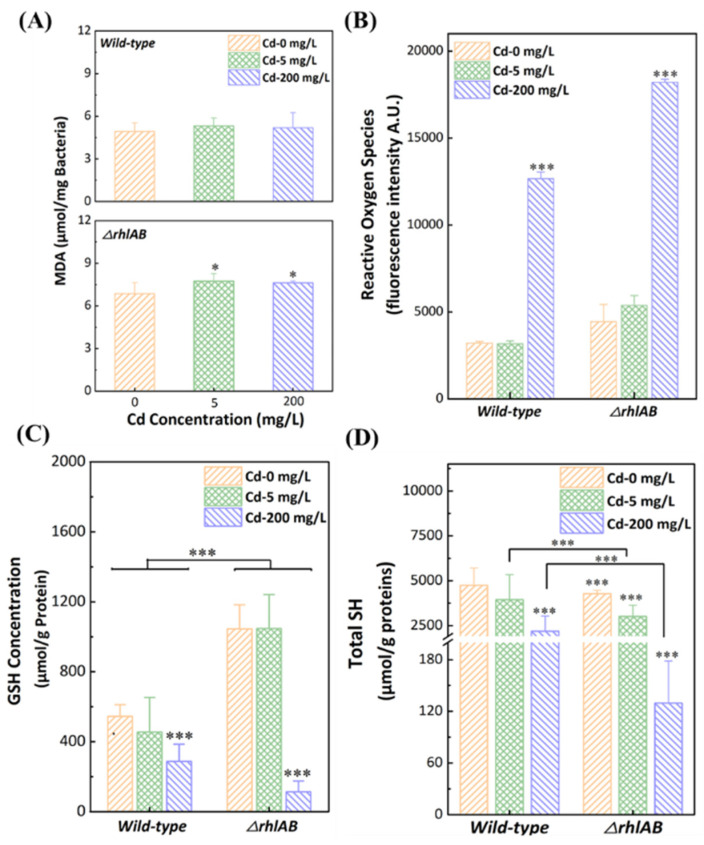
Bacterial membranes damaged (MDA, **A**), reactive oxygen species (ROS, **B**), reductive glutathione (GSH, **C**), and total sulfhydryl group (Total-SH, **D**) amounts for wild-type and Δ*rhlAB* strains. Differences were considered significant at *p* < 0.05, *** presented *p* < 0.005.

MDA is a byproduct of bacterial membrane oxidation that reflects the degree of peroxidation of membrane lipids under oxidative stress [44,45]. As shown in Figure 3A, there was no obvious change in the wild-type with or without Cd^2+^. However, MDA levels were significantly increased in the Δ*rhlAB* strain in the presence of Cd^2+^ (*p* < 0.05), suggesting that more severe membrane damage occurred in the Δ*rhlAB* strain. These results indicated that the *rhlAB* gene might contribute to the protection of bacterial membrane integrity. In addition, ROS levels increased obviously in both strains at 200 mg/L Cd^2+^, whereas the Δ*rhlAB* strain exhibited a much higher ROS level than the wild-type strain (Figure 3B). This indicated that the Δ*rhlAB* strain was exposed to higher levels of Cd^2+^ due to more intracellular Cd^2+^. As shown in Figure S4, there were no obvious changes in Ca^2+^/Mg^2+^ ATPase and K^+^/Na^+^ ATPase activities in the wild-type strain with the presence of Cd^2+^, but the activities in the Δ*rhlAB* strain with Cd^2+^ decreased significantly, leading to a decrease in the ATP level in cells [46]. Generally, the suppression of energy metabolism could function as a control mechanism to reduce the generation of ROS [47].

Glutathione (GSH) is a tripeptide molecule that still widely existed in bacteria, which is important in the antioxidant defense system and effectively prevents oxidative stress from ROS [48]. Compared to wild-type strains, a higher GSH level was detected in Δ*rhlAB* strains in the absence or presence of 5 mg/L Cd^2+^ (Figure 3C). For cells exposed to 200 mg/L Cd, GSH levels for wild-type and Δ*rhlAB* strains decreased to 288.4 μmol/g protein, and 113.7 μmol/g protein, respectively. It indicated an inadequate resistance ability of Δ*rhlAB* strains for more serious oxidative stress at higher Cd^2+^ concentrations. These results confirmed that rhamnolipids could obviously enhance bacterial resistance to Cd^2+^ at low dosages and the enhancement was limited at high Cd^2+^ stress.

In *P. aeruginosa* cells, cysteine-rich metallothionein proteins (MT) with cysteine residues, calculated by total sulfhydryl groups (total-SH), play an important role in the bioremediation of heavy metals contamination [49]. In this study, total-SH contents were measured to confirm that the wild-type strain exhibited a higher ability to alleviate heavy metal stress. For the wild-type strain, the amounts of total-SH without Cd^2+^ (4746.3 μmol/g protein) or with 5 mg/L Cd^2+^ (3946.1 μmol/g protein) were similar and decreased obviously with 200 mg/L Cd^2+^ (2183.7 μmol/g protein) (Figure 3D). Additionally, the total-SH content at 200 mg/L Cd^2+^ decreased sharply to 129.6 μmol/g protein in the Δ*rhlAB* strain. This was mainly attributed to more Cd^2+^ entering the cells of the Δ*rhlAB* strain and combining with sulfhydryl groups, which was consistent with the distribution of Cd^2+^ in the cells.

In this work, we observed the protective mechanisms of *P. aeruginosa* from the Cd^2+^ through the bacterial membrane into the cytoplasm. When Cd^2+^ stress was not high enough to kill cells, the Cd^2+^ efflux systems could protect the cells from Cd^2+^, resulting in an unconspicuous change in the bacterial antioxidant systems (Figure 3) [28,50,51]. When the Cd^2+^ load was high, sulfhydryl complexed with intracellular Cd^2+^ in MT, which could relieve the cells from the high Cd^2+^ load. Meanwhile, due to the high Cd^2+^ load, more ROS were generated, which could not be completely removed by GSH, promoting membrane depolarization, leading to more severe cell damage. It was worth noting that rhamnolipids could bind with Cd^2+^ to prevent Cd^2+^ from entering the cells, and thus reduced the Cd^2+^ load on the cells.

### 3.5. Effect of Cd^2+^ on Secretion of Rhamnolipids

**Figure 4:**
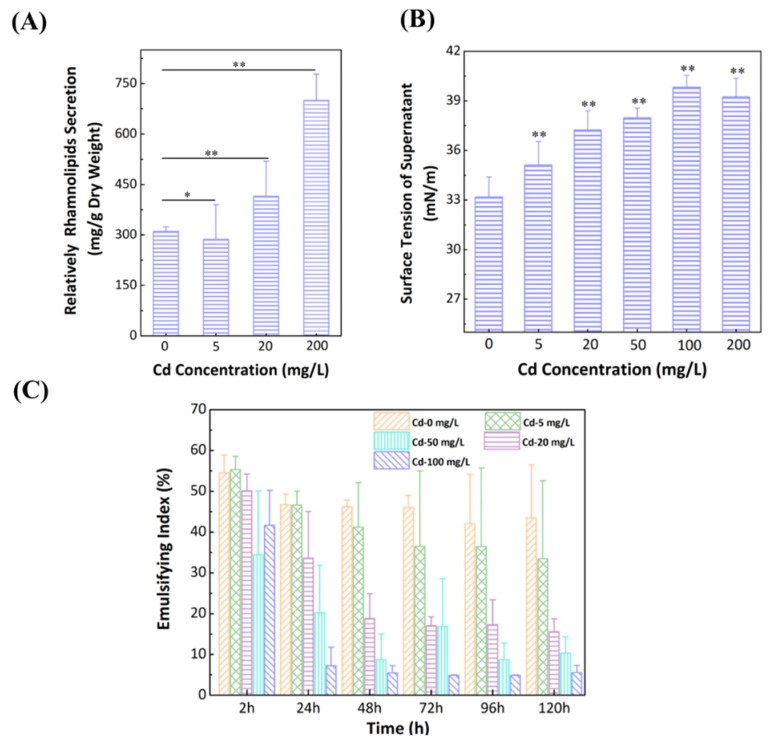
Rhamnolipids secretion (**A**), surface tensions (**B**), and emulsification ability tests (**C**) for the wild-type strain with different Cd concentrations. Differences were considered significant at *p* < 0.05, and * presented *p* < 0.05, ** presented *p* < 0.01.

The secretion of rhamnolipids from the wild-type strain were assessed with a range of Cd^2+^ concentrations. As shown in Figure 4A, Cd stress obviously promoted the production of rhamnolipids per dry biomass, which increased by 143% at 200 mg/L compared with that without Cd^2+^. It has been reported that heavy metal cations can form a complex in a binding pocket, consisting of a carboxylate moiety in the fatty lipid and the hydroxy moiety in the rhamnose [52]. In this study, heavy metal stress affected the secretion of rhamnolipids as the extracellular polymeric substances and thus promoted complex formation with Cd^2+^, which had a beneficial effect of alleviating heavy metal stress [16].

In this experiment, the physicochemical properties of rhamnolipids were studied based on emulsification activity and surface tension. The secretion of rhamnolipids decreased the surface tension (33.2 mN/m) of media without Cd^2+^, compared to the fresh medium (47.5 ± 0.5 mN/m) (Figure 4B). In addition, the increas in Cd^2+^ concentration enhanced surface tensions (35.1, 37.2, and 39.2 mN/m for 5, 20, and 200 mg/L Cd^2+^, respectively). It indicated that Cd^2+^ could impact the stability of the secreted rhamnolipids. The emulsification activities of the biosurfactants were measured with a range of water-immiscible substrates, and the emulsion ability was found to be with hydrocarbons. Secreted rhamnolipids without Cd^2+^ were capable of stabilizing emulsions with an emulsification index (EI) in the range of 46.7% at 24 h to 43.5% at 120 h, whereas the EI value dropped gradually dropped to 4.7% at 100 mg/L (Figure 4C). The low emulsification activity implied that the toxicity of Cd^2+^ to cells, leading to a lower ability to remove hydrophobic organic compound–heavy metal complex contaminations [53].

The chemical structures of the rhamnolipids with Cd were analyzed by FTIR (Figure S5). The peaks were assigned to hydroxyl, rhamnose, and lipid backbones, the main characteristics of glycolipids. Fewer spectral bands in the presence of Cd^2+^ (Table S3) indicated that lipids functional groups appeared to be less or that the fatty acid chains became shorter under Cd^2+^ stress. The absorption intensities of the spectral bands in the presence of Cd^2+^ were weaker than that of the Cd-null sample, as the decreased intensity for the strong and broad bonds of the free-stretching hydroxyl (O-H) due to it complexation with Cd^2+^.

To further investigate the effect of Cd^2+^ loading on the rhamnolipid secretion, we also examined the changes in CMC of rhamnolipids in the presence of Cd^2+^. The CMC values of rhamnolipids were 154.9 mg/L, 269.2 mg/L, and 671.4 mg/L for the cells cultured under Cd^2+^ concentration of 0, 5, and 200 mg/L, respectively (Figure 4A–C). The CMC value of rhamnolipids relates to superficial characteristics, such as surface tension and emulsification activity. However, the value depends on the structure of rhamnolipids, e.g., chain length, number of rhamnoses, etc. [33,54].

Nitschke et al. reported that the major components of rhamnolipids are RhaC_10_C_10_ and Rha_2_C_10_C_10_ in *P. aeruginosa* [55]. However, the composition of rhamnolipids mixtures differs by the presence of unsaturated bonds, fatty acid chains, and the size of hydrophilic groups, which affect the surface character of the mixture. The length of the fatty acid chains and the number of rhamnose units played an important role in the CMC value, affecting hydrophobicity and emulsifying activity [31,56]. Therefore, we concluded that Cd^2+^ actively interacted with rhamnolipids and altered the structural and functional properties of rhamnolipids, which were affected by cultural conditions [57,58]. Our results suggested that Cd^2+^ stress led to shorter fatty acid chains of rhamnolipids, a higher ratio of di-rhamnolipids to mono-rhamnolipids, and a lower proportion of hydrophobic groups, resulting in higher hydrophilicity and lower emulsifying activity and higher concentration is needed to form the micelle aggregation [25].

Rhamnolipids biosynthesis pathway begins with the de novo synthesis of rhamnose and fatty acids, which are ubiquitous in the bacterium followed by three consecutive enzymatic reactions specific in rhamnolipids-secreting bacterium. Our results suggested that Cd stress effects *rhlA, rhlB,* and *rhlC* expression (Figure S6) and rhamnolipids might also be a endogenous factor in response to heavy metals stress. A strong correlation between rhamnolipids production and heavy metal stress has also been documented for other *P. aeruginosa* strains. The ratio of di-rhamnolipids to mono-rhamnolipids increased obviously under Cd^2+^ stress [32]. This may explain the increase of bacterial surface hydrophilicity in the presence of Cd^2+^ in this study.

**Figure 5:**
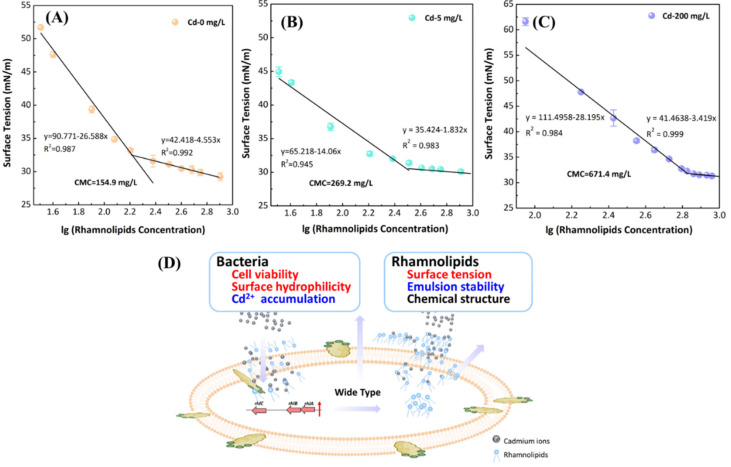
Critical micelle concentrations (**A**, **B**, and **C** for 0, 5, and 200 mg/L Cd^2+^, respectively) of rhamnolipids extracted from wild-type cells with different Cd concentrations; schematic diagram for the effect of Cd^2+^ on wild-type bacterial cells and rhamnolipids; words in red represent enhancement, blue words represent reduction (**D**).

Briefly, the interactions among bacteria, endogenous rhamnolipids, and cadmium were demonstrated in Figure 5D as follows: (1) the endogenous rhamnolipids with more rhamnose tails could form a complex with additional cadmium, reduce intracellular Cd^2+^ accumulation, decrease oxidative stress response, and improve bacterial cell viability; (2) the altered physicochemical properties of endogenous rhamnolipids, such as increased surface tension and decreased emulsification activity, was attributed to the changed structures of rhamnolipids under Cd^2+^ stress; and (3) the altered properties of rhamnolipids resulted in a more hydrophilic bacterial surface. These changes negatively affected the utilization of endogenous rhamnolipids.

## 4. Conclusions

Biosurfactants secreted by microorganisms can probably improve the removal of contaminants and reduce environmental hazards. Together with our results, this study confirmed the potential of employing rhamnolipids and rhamnolipids-secreted bacteria in Cd^2+^ remediation. Cadmium stress could affect rhamnolipid production and increase the complexation of Cd^2+^ on bacterial surfaces, resulting in the protection of cells from Cd^2+^ stress. For the wild-type cells, Cd stress altered the structure of rhamnolipid structures, leading to higher CMC and hydrophilicity, and this discrimination prejudiced emulsification ability. Once Cd entered the cytoplasm, the oxidative stress system had no obvious effects on the membrane integrity of the wild-type due to other stress mitigation mechanisms available, such as GSH, total-SH, ATPase activity, cation efflux pump, etc. For the mutant cells, the lack of rhamnolipid secretion led to greater intracellular Cd accumulation, which motivated fearful oxidative stress that could not be alleviated by detoxification mechanisms and impaired cell viability as a result. As with higher Cd loading, intracellular Cd accumulation increased; however, the cell viability of the wild-type was slightly higher than that of the mutant. The GSH content and total-SH content for the mutants were much lower than in the wild-type cells. These findings suggested that rhamnolipids had a limited effect on improving Cd^2+^ resistance. This study enriched the theoretical knowledge on the use of *P. aeruginosa* to enhance the bioremediation of heavy metals bioremediation and alleviate stress in contaminated environments.

## Data Availability

All data used in this study are included in this published article and its supplementary information file.

## Supplementary Materials

The following are available online at https://www.mdpi.com/article/10.3390/ijerph191912555/s1, Figure S1. The schematic of pEX18GM-△*rhlAB* plasmid construction. Figure S2. DNA gel electrophoresis (A), rhamnolipids secretions (B), and CTAB-MB plate (C) to verified the △*rhlAB* strain could not secreted rhamnolipids. Figure S3. Distribution of cadmium for the wild-type and the △*rhlAB* strains under different Cd^2+^ concentrations (5, 200 mg/L). Figure S4. ATPase activity (Na^+^/K^+^, and Ca^2+^/Mg^2+^) assays for the wild-type and the △*rhlAB* strains which expressed in U per mg proteins. Figure S5. ATR-FTIR assays for semi-purified rhamnolipids from the wild-type under a series of Cd^2+^ concentrations (0, 5, 50, 100, 200 mg/L). Figure S6. Expression of genes of the wild type under the stress of Cd. Table S1. Plasmids and bacteria used in this study. Table S2. The primers and sequences for *rhlA-rhlB* deletion and qPCR. Table S3. Assignments of FTIR spectra of rhamnolipids. References [32,59,60,61,62] are cited in the supplementary materials
