# Supplementary material for: Limited Role of Rhamnolipids on Cadmium Resistance for an Endogenous-Secretion Bacterium
Source: Int J Environ Res Public Health. 2022 Oct 1;19(19):12555. doi: 10.3390/ijerph191912555 (PMC9566264; doi:10.3390/ijerph191912555)
Supplement: Supplementary file 1 [file ijerph-19-12555-s001.zip › ijerph-1945510-supplementary.pdf]

Supplementary Information for
Limited role of rhamnolipids on cadmium resistance for an endogenous-secretion bacterium

Sufang Xing[1], Zhen Yan[1], Chao Song[1], Huifang Tian[1], and Shuguang Wang[1,2*]

1 Shandong Key Laboratory of Water Pollution Control and Resource Reuse, School of Environmental Science and Engineering, Shandong University, Qingdao 266237, China
2 Sino-French Research Institute for Ecology and Environment (ISFREE), School of Environmental Science and Engineering, Shandong University, Qingdao 266237, China

*Corresponding author Shu-Guang Wang
E-mail: wsg@sdu.edu.cn

Blue agar test. Blue agar could be used to performing to detect the secretion of biosurfactants by minimal agar medium containing 0.04% cetyl trimethyl ammonium bromide (CTAB), and 0.02% methylene blue [59]. 2.5 μL of media was added to blue agar plate and incubated for 72 h at 37 °C. The presence of biosurfactants was confirmed by the development of deep blue color halos around the colony.

Surface tension measurement. Bacterial cells cultivated in LB media with series of Cd^{2+} concentrations, and reached stable period. Supernatant harvested from bacterial media by centrifugation.

Emulsifying activity. Emulsification was used to measure the effectiveness of biosurfactants. Emulsifying activity was determined by mixing the bacterial-free supernatants with equal amounts of hydrophobic solvents (paraffin oil), vortexed the mixtures and static setting at room temperature. Bacterial-free supernatants obtained from range of Cd^{2+} concentrations (0, 5, 20, 50, 200 mg/L). The emulsification index (EI) was measured each 24 h, which determined as follows [60]:

EI=(Height of the emulsified layer)/(Total height of the liquid column)×100% (1)

Critical Micelle Concentration. Determination of critical micelle concentration (CMC) of semi-purified rhamnolipids were performed in aqueous solution.

FTIR Assay. The molecular structure of semi-purified rhamnolipids were measured by FT-IR spectra. The extracted rhamnolipids from different bacterial media were completely dried to powder by freeze drying, and analysis using a spectrum (Nicolet iS50). The measurement condition was chosen as follows: the spectral range from 4000 cm^{-1} to 400 cm^{-1}, 32 scans with 4 cm^{-1} resolution [61].

Quantitative real-time PCR Assay. Quantitative real-time PCR (RT-qPCR) was used to measure the expressions of the three related genes to rhamnolipids synthesis (*rhlA*, *rhlB* and *rhlC*) in *P. aeruginosa* under Cd stress. Bacteria cultivated by LB media with different concentrations of Cd^{2+} (0, 20, 200 mg/L). In this study, 16S rRNA was used as the housekeeping gene. For each sample, a threshold cycle value (CT) was determined, and results were analyzed with the comparative Ct method ($2^{-\Delta\Delta Ct}$) [32,62]. Total RNA was isolated from collected bacterial cells according to the standard protocol. The quantity and quality of RNA samples were assessed with Thremo NanoDrop One Spectrophotometer (Thermo Scientific). Subsequently, total RNA was reverse transcribed using M5 First Strand cDNA Synthesis Kit (Mei5 Biotechnology. Co., Ltd, Beijing, China). Details of this experiment have shown in *Supporting Information*. The primers for RT-qPCR were designed as showed in Table S2.

For the total RNA extraction, the bacterial cultures (10 mL for each) were sampled at the stable phase. Total RNA was extracted using M5 EASYspin Plus Bacterial RNA Extraction Kit (Mei5 Biotechnology. Co., Ltd, Beijing, China) according to the instructions. Cultivation samples were prepared for three replicates. The experiments were performed by the Realtime PCR Super mix (SYBR green, with anti-Tap) manufacturer's instructions. Thermal cycle sited as on cycle of 95 °C for 1 min, followed by 40 cycles of 95 °C for 15 s, 57.5 °C for 30s, and 72 °C for 15 s, followed by melt curve analysis. All amplifications were performed in triplicate. The total PCR volume was 20 μL, each PCR run included a control well containing ddH_2O for negative control for each gene. The reaction efficiencies were calculated using the equation $E=10^{(-1/slope)}$ from the slope was measured using 5-fold gradient dilution. Reaction efficiencies of all primers were above 90% in this experiment.

Figure S1 The schematic of pEX18GM-△*rhlAB* plasmid construction.
Figure S2 DNA gel electrophoresis (A), rhamnolipids secretions (B), and CTAB-MB plate (C) to verified the △*rhlAB* strain could not secreted rhamnolipids.

Figure S3 Distribution of cadmium for the wild-type and the △*rhlAB* strains under different Cd^{2+} concentrations (5, 200 mg/L).

Figure S4 ATPase activity(Na^+/K^+, and Ca^{2+}/Mg^{2+}) assays for the wild-type and the △*rhlAB* strains which expressed in U per g proteins.

Figure S5 ATR-FTIR assays for semi-purified rhamnolipids from the wild-type under a series of Cd^{2+} concentrations.

Figure S6 Expression of genes of the wild-type under the stress of Cd.

Table S1 Plasmids and bacteria used in this study.
Table S2 The primers and sequences for *rhlA-rhlB* deletion and qPCR.
Table S3 Assignments of FTIR spectra of rhamnolipids.

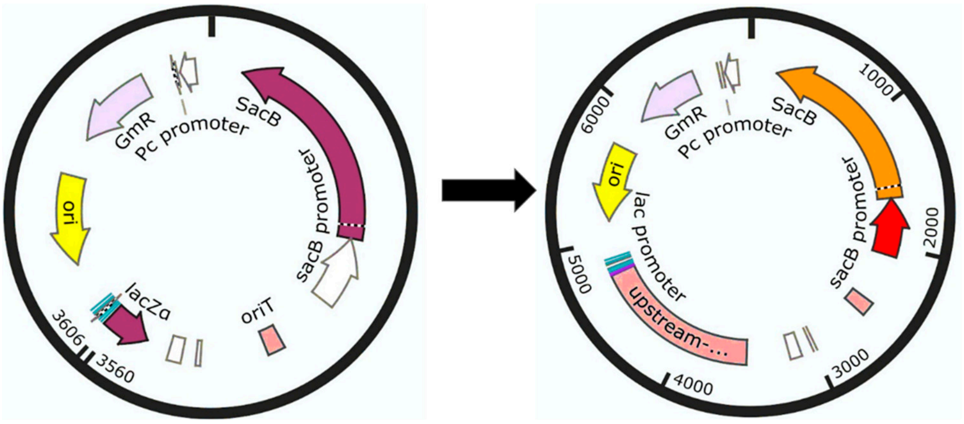

Figure S1 The schematic of pEX18GM-△*rhlAB* plasmid construction.

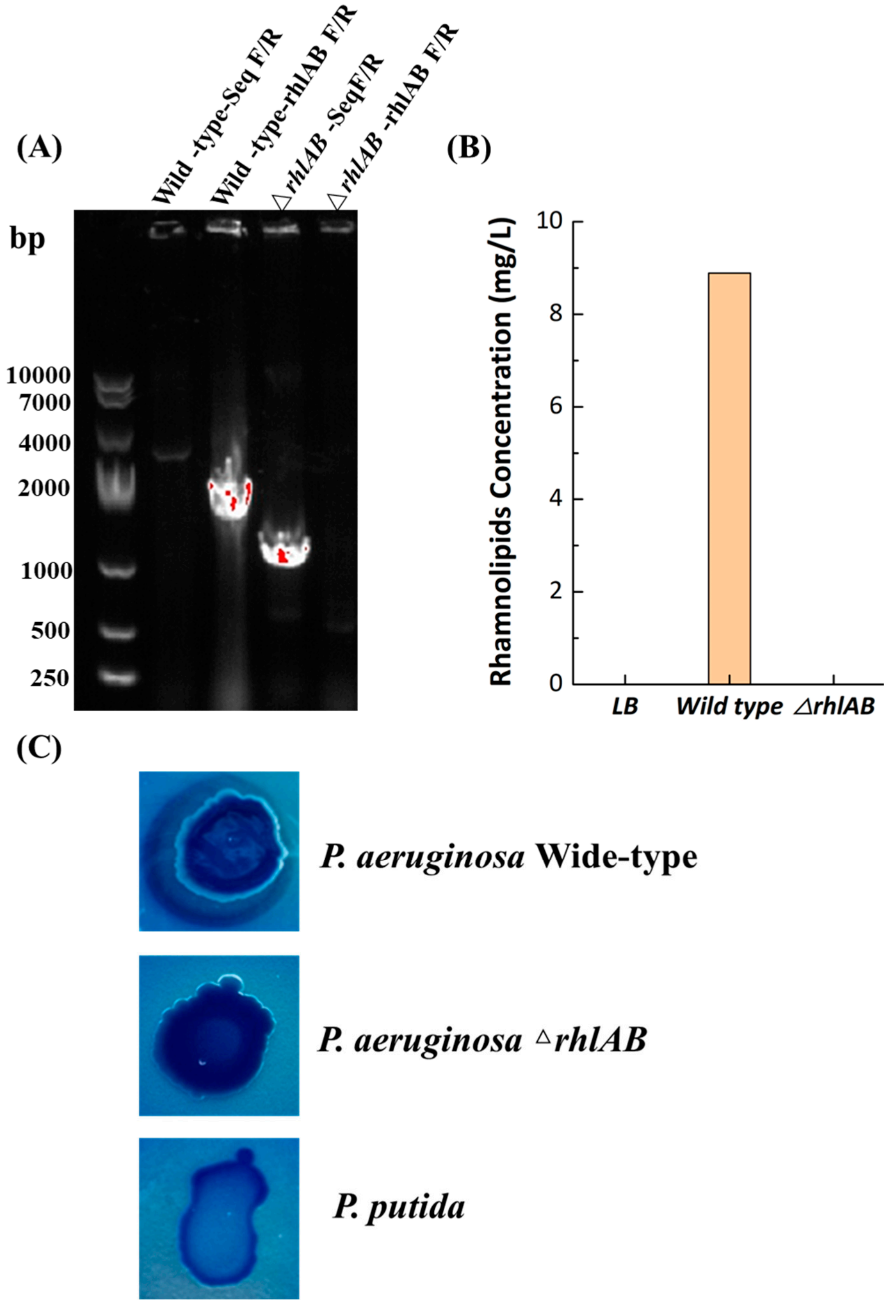

Figure S2 DNA gel electrophoresis (A), rhamnolipids secretions (B), and CTAB-MB plate (C) to verified the △*rhlAB* strain could not secreted rhamnolipids.

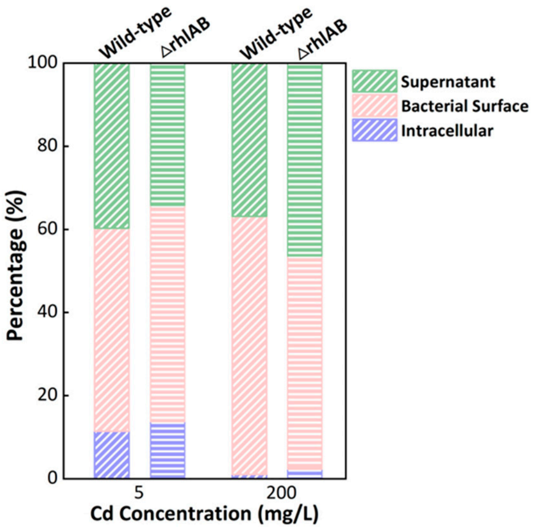

Figure S3 Distribution of cadmium for the wild-type and the △*rhlAB* strains under different Cd^{2+} concentrations (5, 200 mg/L).

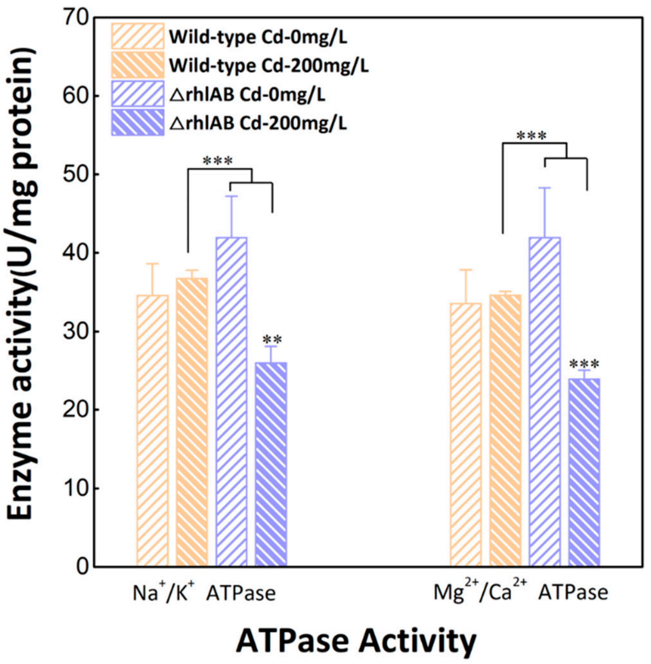

Figure S4 ATPase activity (Na^+/K^+, and Ca^{2+}/Mg^{2+}) estimation for the wild-type and the △*rhlAB* strains which expressed in U per mg proteins.

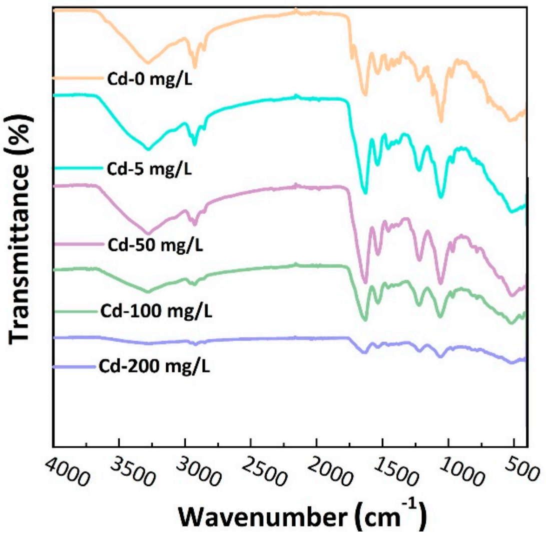

Figure S5 ATR-FTIR assays for semi-purified rhamnolipids from the wild-type under a series of Cd^{2+} concentrations (0, 5, 50, 100, 200 mg/L).

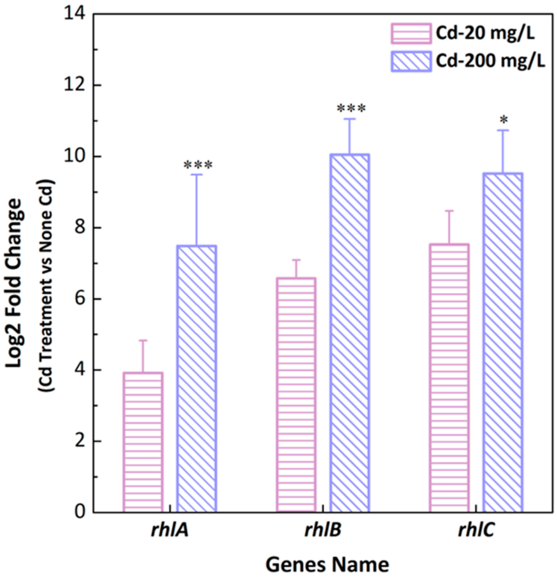

Figure S6 Expression of genes of the wild-type under the stress of Cd.

Table S1. Plasmids and bacteria used in this study.

Name	Description and pertinent features
Bacteria	
P. aeruginosa	Wild type
E. coli MW3084	With pEX18GM
E. coli MW3084 △*rhlAB*	In this study
Vector	
pEX18GM	Gm^r, *oriT*, *sacB*, *lacZα*, MCS from pUC18
pEX18GM △*rhlAB*	In this study

Table S2 The primers and sequences for *rhlA-rhlB* deletion and qPCR.

**Primers**	**Sequence (5′→3′)**
XSF-LF	GAATTCGTAATCATGGTCATAGCTGTTTCC
XSF-LR	AAGCTTGGCACTGGCCGTC
XSF-UF	GCCAGTGCCAAGCTTTGACCCTCGAGTTCTCCAAT
XSF-UR	CAGCACCGTTCAGGACGCGCGCCGCATAAAAATTTT
XSF-DF	GCTGCGTCCTGAACGGTGCTG
XSF-DR	CATGATTACGAATTCCCGCAGGCGGATTTCCT
XSF-UDF	GCCAGTGCCAAGCTTTGACCCTCGAGTTCTCCAAT
XSF-UDR	CATGATTACGAATTCCCGCAGGCGGATTTCCT
rhlA-F	AGACCGTCGGCAAATACCTG
rhlA-R	TGCCGTTGATGAAATGCACG
rhlB-F	CCGGTACACCCCAAGTTCAA
rhlB-R	GGGATACTGCCGTCGAACAG
rhlC-F	ATCCATCTCGACGGACTGAC
rhlC-R	CGGAGGAGATCAGGAACGAG
16S-341F	CCT ACG GGA GGC AGC AG
16S-518R	ATT ACC GCG GCT GCT GG

Table S3 Assignments of FTIR spectra of rhamnolipids.

**Wavenumber(cm^{-1})**		**Assignments**
**Common**	3270-3277	hydroxyl group free stretch due to H-bond interaction between groups of rhamnolipids
	2970-2850	C-H stretching vibrations of biosurfactants preformed the hydrocarbon sites in backbone
	1627	carbonyl stretching with strong-intensity bands
	1535	$-COO^-$
	1455-1370	aliphatic bonds stretching
	1300-1033	C-O stretch confirmed the bonds between carbon and hydroxyl groups in the structures of rhamnose
	3026-3084	hydroxyl group
**Different**	1729	carbonyl stretching with strong-intensity bands
	1319	aliphatic bonds twisting for fatty acids bands
	1166	C-C symmetric stretching for lipids or polysaccharides
